# Defibrillate You Later, Alligator: Q10 Scaling and Refractoriness Keeps Alligators from Fibrillation

**DOI:** 10.1093/iob/obaa047

**Published:** 2021-01-27

**Authors:** Conner Herndon, Henry C Astley, Tomasz Owerkowicz, Flavio H Fenton

**Affiliations:** 1 School of Physics, Georgia Institute of Technology, Atlanta, GA, USA; 2 Department of Biology, Biomimicry Research & Innovation Center, University of Akron, Akron, OH, USA; 3 Department of Biology, California State University, San Bernardino, CA, USA

## Abstract

Effective cardiac contraction during each heartbeat relies on the coordination of an electrical wave of excitation propagating across the heart. Dynamically induced heterogeneous wave propagation may fracture and initiate reentry-based cardiac arrhythmias, during which fast-rotating electrical waves lead to repeated self-excitation that compromises cardiac function and potentially results in sudden cardiac death. Species which function effectively over a large range of heart temperatures must balance the many interacting, temperature-sensitive biochemical processes to maintain normal wave propagation at all temperatures. To investigate how these species avoid dangerous states across temperatures, we optically mapped the electrical activity across the surfaces of alligator (*Alligator mississippiensis*) hearts at 23°C and 38°C over a range of physiological heart rates and compare them with that of rabbits (*Oryctolagus cuniculus*). We find that unlike rabbits, alligators show minimal changes in wave parameters (action potential duration and conduction velocity) which complement each other to retain similar electrophysiological wavelengths across temperatures and pacing frequencies. The cardiac electrophysiology of rabbits accommodates the high heart rates necessary to sustain an active and endothermic metabolism at the cost of increased risk of cardiac arrhythmia and critical vulnerability to temperature changes, whereas that of alligators allows for effective function over a range of heart temperatures without risk of cardiac electrical arrhythmias such as fibrillation, but is restricted to low heart rates.

**Synopsis** La contracción cardíaca efectiva durante cada latido del corazón depende de la coordinación de una onda eléctrica de excitación que se propaga a través del corazón. Heterogéidades inducidas dinámicamente por ondas de propagación pueden resultar en fracturas de las ondas e iniciar arritmias cardíacas basadas en ondas de reingreso, durante las cuales ondas espirales eléctricas de rotación rápida producen una autoexcitación repetida que afecta la función cardíaca y pude resultar en muerte súbita cardíaca. Las especies que funcionan eficazmente en una amplia gama de temperaturas cardíacas deben equilibrar los varios procesos bioquímicos que interactúan, sensibles a la temperatura para mantener la propagación normal de ondas a todas las temperaturas. Para investigar cómo estas especies evitan los estados peligrosos a través de las temperaturas, mapeamos ópticamente la actividad eléctrica a través de las superficies de los corazones de caimanes (*Alligator mississippiensis*) a 23°C and 38°C sobre un rango de frecuencias fisiológicas del corazón y comparamos con el de los conejos (*Oryctolagus cuniculus*). Encontramos que a diferencia de los conejos, los caimanes muestran cambios mínimos en los parámetros de onda (duración potencial de acción y velocidad de conducción) que se complementan entre sí para retener longitudes de onda electrofisiológicas similares a través de los rangos de temperaturas y frecuencias de ritmo. La electrofisiología cardíaca de los conejos acomoda las altas frecuencias cardíacas necesarias para mantener un metabolismo activo y endotérmico a costa de un mayor riesgo de arritmia cardíaca y vulnerabilidad crítica a los cambios de temperatura, mientras que la de los caimanes permite un funcionamiento eficaz en una serie de temperaturas cardíacas sin riesgo de arritmias eléctricas cardíacas como la fibrilación, pero está restringida a bajas frecuencias cardíacas.

## Introduction

Variations in temperature can have large effects on the dynamics of many chemical processes, including those underlying biological systems (Hegarty 1973). A decrease in temperature can dramatically lower rate-dependent processes such as muscle power and activation time, heart rate, immune function, and digestive rate (Bennett 1985; Driedzic and Gesser 1994; Le Morvan et al. 1998; Wang et al. 2002). These reaction rate changes can also lead to changes at much higher scales, including whole-animal performance, behavior, ecology, and phenology (Greenwald 1974; Bennett and John-Alder 1984; Marsh and Bennett 1986; Waldschmidt et al. 1986; John-Alder et al. 1988). However, these variations due to temperature changes are not equal across processes or species, as changes of 10°C can lead to losing performance rapidly for some, while others can have minimal performance loss, even across a broad range of temperatures (Bennett 1985; James 2013).

For the heart, cardiac function across temperatures presents a particularly interesting problem due to complex interactions between multiple physiological processes. Effective cardiac function relies on rhythmic contraction, which is orchestrated by a coordinated propagation of electrical excitation across the heart. Wave dynamics emerge from complex ionic exchange both across the membrane and within cells (Hodgkin and Huxley 1952; Fenton and Cherry 2008), and the reaction rates of underlying physiological processes affect wave speed (Veeraraghavan et al. 2014), refractory period, and action potential duration (APD; Mines 1913). The propagation of an action potential across the heart is followed by a spatially extended region of refractory tissue, within which attempted re-excitations fail to evoke an action potential. The spatial wavelength of an action potential and its subsequent refractory region safeguard against reentrant-based arrhythmia, wherein a fragmented excitation wavefront forms a spiral wave of electrical activity (Davidenko et al. 1992; Cherry and Fenton 2008) that has the potential to repeatedly turn and re-excite post-refractory tissue (Mines 1913; Allessie et al. 1976, 1977). Dynamically induced heterogeneities in refractoriness due to complex wave dynamics (Qu et al. 2000; Watanabe et al. 2001) can lead to wavebreak and the initiation of multiple re-entrant spiral waves (Fenton et al. 2002) which produce irregular spatiotemporal dynamics that compromise cardiac function and drive the heart toward ventricular fibrillation (VF) and sudden cardiac death (Gray et al. 1998).

One of the ways species may accommodate increased metabolic demand is through an increased heart rate (i.e., a reduced cardiac cycle length). This reduction in time that the heart may receive and eject blood introduces concomitant challenges that necessitate a variety of anatomical and physiological modifications, many of which are themselves paired with trade-offs and consequences (Burggren et al. 2014). One such requirement for a reduced cardiac cycle length is an accompanying reduction of the APD, which exhibits cycle length-dependent shortening known as restitution (Elharrar and Surawicz 1983). If the action potential wavelength (the safeguard against reentrant-based arrhythmia) shrinks to a spatial extent comparable to the heart, there is a risk of sudden cardiac death (Mines 1913; Allessie et al. 1976, 1977; Klebér and Rudy 2004). The cardiac electrophysiology in species which demand high metabolic performance is therefore challenged to coordinate its variation in wave properties across cardiac cycle lengths to maintain sufficiently large action potential wavelengths while minimizing the risk of arrhythmia.

Temperature affects heart rate across many species (Wilber 1960; DeFur and Mangum 1979; Driedzic and Gesser 1994; Lillywhite et al. 1999). If different physiological processes show different Q_10_ values, these crucial parameters may become mismatched and adversely affect coordinated wave dynamics as temperature changes. These risks are further exacerbated by Q_10_ values which themselves may vary across heart rates (Shah et al. 2006). In mammals, these temperature-induced mismatches can lead to wave instabilities and fibrillation at low temperatures (Smeets et al. 1986; Fenton et al. 2013; Filippi et al. 2014), but many poikilothermic (“cold-blooded”) animals must operate across a wide range of core body temperatures and, within each temperature, at a wide range of heart rates (from rest to maximal exertion). Consequently, we hypothesize that species that must function effectively across a range of heart temperatures will display lower thermal sensitivity of cardiac wave properties and a broader range of heart rates without unstable or deleterious wave patterns compared to species that maintain constant heart temperature. Locomotor muscles of poikilothermic species with a preference for higher body temperatures in the wild (Bennett 1985) and metabolically warmed muscles of regional heterotherms (Donley et al. 2007) show greater sensitivity to temperature than muscles of species with preference for cooler and more variable body temperatures; data are too sparse to reach a definite conclusion on whether the same effect holds true in homeothermic endotherms (James 2013). To test this hypothesis, we examine how temperature induces wave property mismatch in domestic rabbits (*Oryctolagus cuniculus*) and juvenile captive American alligators (*Alligator mississippiensis*).

## Methods

### Experimental animals

We quantified the transmembrane voltage dynamics across the hearts of New Zealand white rabbits (*O. cuniculus*, *N* = 3, 2–3 kg, age >6 months) and American alligators (*A. mississippiensis*, *N* = 5, age 4 months, ∼300 g) via optical mapping (Uzelac et al. 2017). Both species have four-chambered hearts of similar size (∼3 cm); however, while rabbits maintain a constant heart temperature of 38°C, the body temperature of active, wild alligators ranges from 10°C to 37°C (Brattstrom 1965; Lewis and Gatten 1985). Unfortunately, only three alligators could be fully analyzed since heart motion could not be suppressed with blebbistatin, an issue that has also been reported elsewhere recently (Jensen et al. 2018). All experimental procedures were approved by the office of Research Integrity Assurance of Georgia Tech under IACUC no. A15034. Alligator eggs were collected at the Rockefeller Wildlife Refuge in Grand Chenier, Louisiana, and incubated (at 30°C) and hatched at CSUSB before being transferred to GT. Alligators were maintained communally in environments with access to water and dry land with ambient temperature maintained at 25°C.

### Anesthesia and euthanasia

Alligators were captured and manually restrained by an experienced handler (HCA) and given an intramuscular injection of ketamine/xylazine (7.5–10/1–2 mg kg^−1^, respectively) for sedation. Once sedated, heparin (300 U kg^−1^) was injected via the occipital sinus, followed by a short delay for circulation and an overdose of sodium pentobarbital (150 mg kg^−1^), after which the heart was removed for experimentation. Rabbits were anesthetized with ketamine/xylazine/acepromazine (17/9/0.9 mg kg^−1^, respectively) and injected with heparin (300 U kg^−1^). After 5 min, euthanasia was induced with pentobarbital (120 mg kg^−1^), and hearts were quickly excised via left thoracotomy.

### Optical mapping

Immediately following extraction, retrograde perfusion via the aorta in rabbits and right (systemic) aorta in alligators with cardioplegic solution (in mM: NaCl 110, KCl 16, NaHCO_3_ 10, MgCl_2_·6H_2_O 16, and CaCl_2_·2H_2_O 1.2) allowed transport to the lab (˂10 min), where hearts were immersed in a temperature-controlled chamber and perfused with Tyrode’s solution (in mM: NaCl 124, KCl 4, NaHCO_3_ 24, NaH_2_PO_4_·H_2_O 0.9, MgCl_2_·6H_2_O 0.7, CaCl_2_·2H_2_O 2, and Dextrose 5.5) gassed with 95% O_2_ and 5% CO_2_ at a pressure of ∼60 mmHg controlled by peristaltic pump. Hearts were stained for 15–20 min with 40 *μ*M of voltage-sensitive dye Di-4-ANBDQPQ (Matiukas et al. 2007), previously dissolved in ethanol (24.4 mg mL^−1^). Heart motion was suppressed by perfusion with 2–5 *μ*M blebbistatin 20–30 min prior to data acquisition. Blebbistatin was ineffective in suppressing contraction in the alligator hearts, which required an increased pressure to further prevent motion of the heart and provide stable images. Transmembrane voltages across the surfaces of the hearts were obtained via fluorescence imaging using an EMCCD camera at a frame rate of 250 or 500 Hz and spatial resolution of 128 × 128 pixels, corresponding to approximately 250 *μ*m pixel^−1^.

### Electrical restitution

Hearts were stimulated with an AgCl bipolar lead current source at the apex separated by ∼1 mm, delivering at least 4× the current necessary to induce excitation. Steady-state cardiac dynamics were recorded over a range of cardiac cycle lengths by following the steady-state restitution pacing protocol (Elharrar and Surawicz 1983), whereby the heart is stimulated at a constant period, referred to as the basic cycle length (BCL), and allowed to equilibrate for a minimum of 30 beats before recording. Hearts were first stimulated at the largest BCL that prevented self-pacing followed by incremental reductions of 50 ms (alligator) and 10 ms (rabbit) until either the ventricular functional refractory period (VFRP) was reached or VF occurred. Experiments were first conducted at $23\pm 1^{{}^{\circ}}$C followed by replication at $38\pm 1^{{}^{\circ}}$C.

### Analysis

**Fig. 1:**
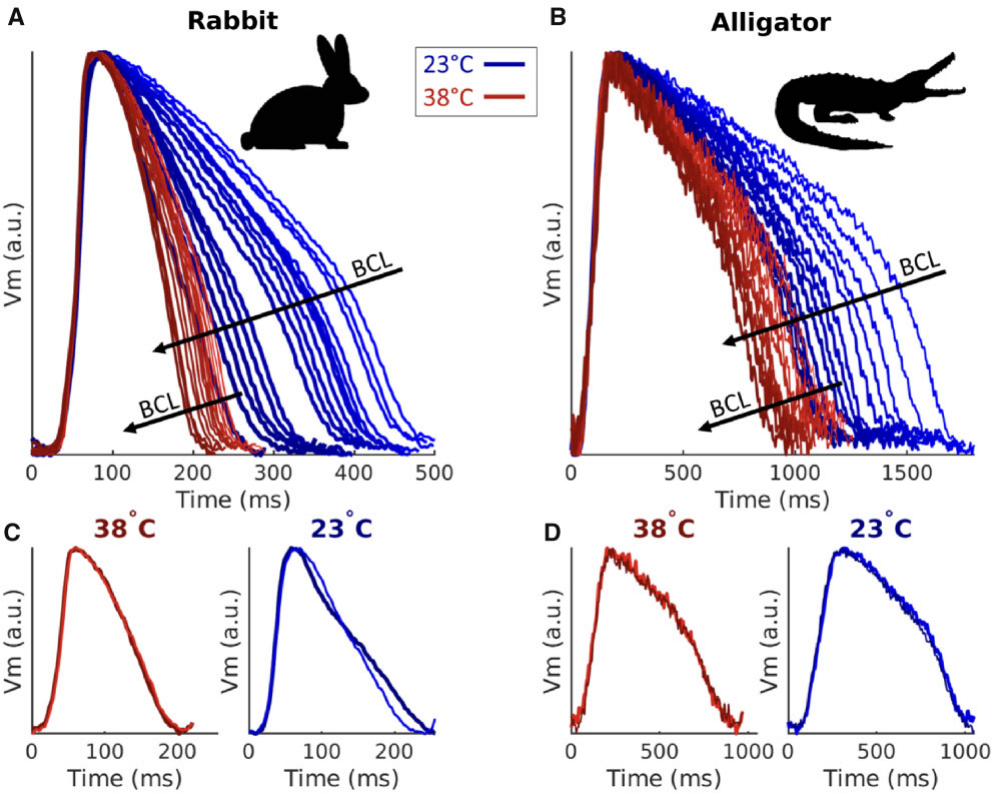
Effect of temperature and BCL on APD and morphology in rabbit (**A** and **C**) and alligator (**B** and **D**). (**A** and **B**) Representative APs for all measured BCLs overlaid to accentuate BCL and temperature dependence. As BCL is shortened, APD likewise decreases. (**C** and **D**) Two subsequent APs from one BCL are overlaid to show beat-to-beat variation in AP morphology at 38°C (left) and 23°C (right). Note that *x*-axis scales are different for the two species.

**Fig. 2:**
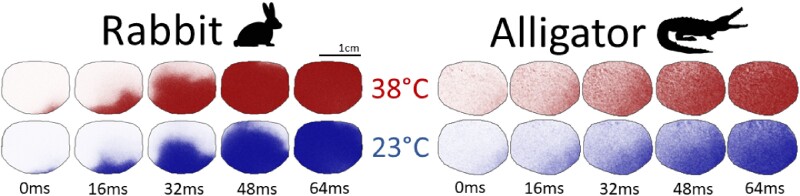
Snapshots of the propagation of an action potential across similar sized regions of tissue in rabbit (left) and alligator (right) at 38°C (top) and 23°C (bottom). The difference in wave propagation between temperatures is nearly indistinguishable in the alligator heart, whereas the rabbit heart displays a clear CV reduction in hypothermia. The wavefront of excitation in the alligator is not as discernible as in the rabbit due to a nearly order of magnitude longer depolarization, which results in a “smearing” of the action potential’s wavefront over the alligator’s heart.

Fluorescent signals were smoothed in time (moving average, window size of two frames) and in space (5 × 5 pixel Gaussian kernel, *σ* = 3 pixels), and signals were detrended by removing linear fits to signal baselines in each pixel. APD was defined as the amount of time that transmembrane voltage (Vm) was maintained >70% repolarization over the course of an action potential. For clarity in comparison between hearts, the values we report for each BCL are the median of APDs following equilibration over a range of 20 × 20 pixels near the apex of each heart with bars signifying one standard deviation (1SD) For a general view of how action potentials varied across BCLs and temperature, a single representative from each is selected and all are plotted together in Fig. 1. Likewise, the qualitative effect of temperature on action potential propagation across the heart is shown in Fig. 2 through snapshots of the heart’s surface over the course of a single action potential.

**Fig. 3:**
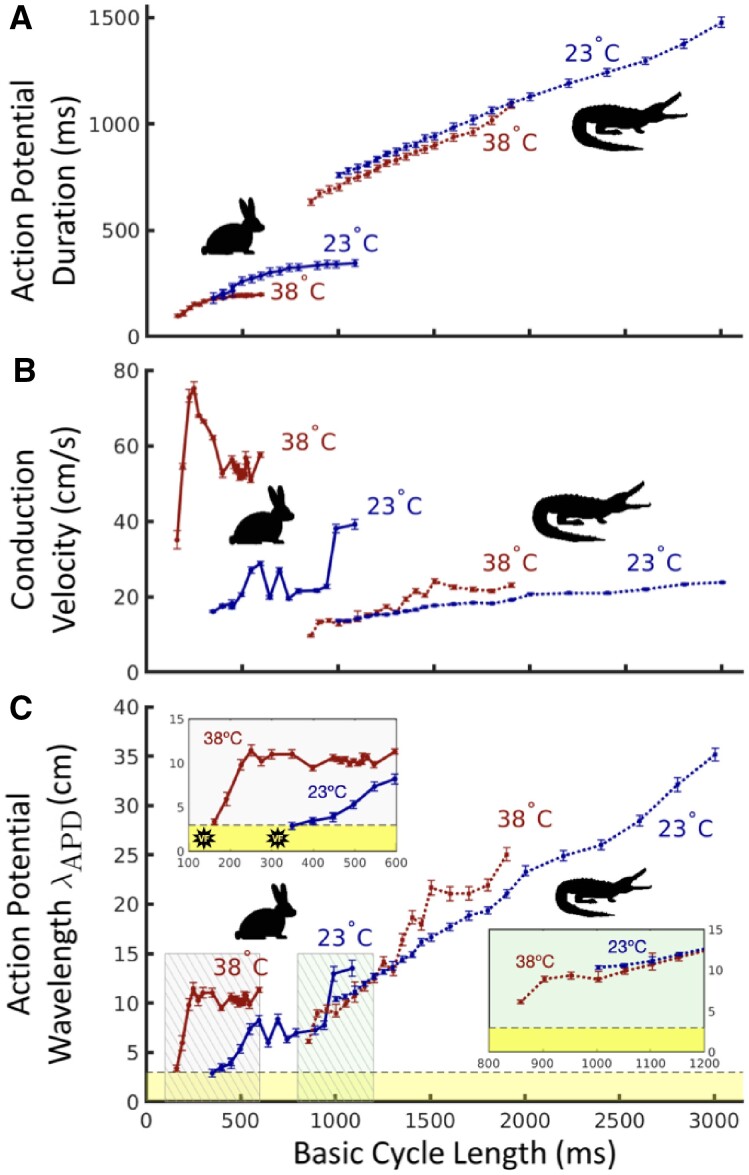
Cardiac electrical wave characteristics in rabbit and alligator at 38°C (red) and 23°C (blue) across BCLs. (**A**) APD. APDs in rabbit exhibit an increased temperature-dependent divergence at larger BCLs, but alligator APDs are consistently altered by temperature across all BCLs. (**B**) CV. Reduction of CV at 23°C is substantial in rabbit but negligible in alligator. (**C**) Action potential wavelength $\lambda_{\text{APD}}$. VF occurred in rabbit hearts when stimulated at BCLs for which $\lambda_{\text{APD}}$ fell below the ventricular length scale (yellow region below horizontal dashed line). The long VFRP in the alligator prohibited $\lambda_{\text{APD}}$ from approaching this critical zone. Insets are magnifications of correspondingly colored regions.

The speed of wave propagation across the heart, or conduction velocity (CV), was calculated by first assigning for each (x,y) coordinate the time at which the corresponding pixel achieved its maximum upstroke velocity ${\text{dV}}_{m}/\text{dt}{|}_{\text{max}}$ (Laughner et al. 2012) over the course of each action potential propagation. We define CV as the inverse of the magnitudes of this surface’s gradient vectors. A corresponding action potential wavelength ($\lambda_{\text{APD}}$) may then be calculated as the product of APD and CV (i.e., $\lambda_{\text{APD}}=\text{APD}\cdot \text{CV}$). Since all hearts were stimulated from the apex, we report only apex-to-base CV and do not report anisotropy ratio, the quotient of longitudinal to transverse CV. For short BCLs, action potentials may exhibit a beat-to-beat alternation in APD (Laurita and Rosenbaum 2008) and CV (Frame and Simson 1988) known as alternans. Data at each BCL were thus separated by even and odd beats and analyzed separately to account for alternans. For clarity, we report only the set of action potentials exhibiting the lower value of APD and CV in the figures, as our focus is on the risk of arrhythmia brought about by the shortening of action potential wavelength. Values for APD, CV, and $\lambda_{\text{APD}}$ are shown in Fig. 3 for a single rabbit and alligator (others are provided in Supplementary Material).

The impact of temperature on tissue scale parameters such as APD and CV can be quantified by a temperature coefficient *Q*_10_ (Shah et al. 2006). The *Q*_10_ value associated with some measured dynamic property K is defined such that its values *K*${}_{T_{1}}$ and *K*${}_{T_{2}}$ at temperatures T_1_ and T_2_, respectively, are related by

$$K_{T_{2}}=K_{T_{1}}\cdot Q_{10}{}^{\left(T_{2}-T_{1}\right)/10^{{}^{\circ}}C}.$$

**Fig. 4:**
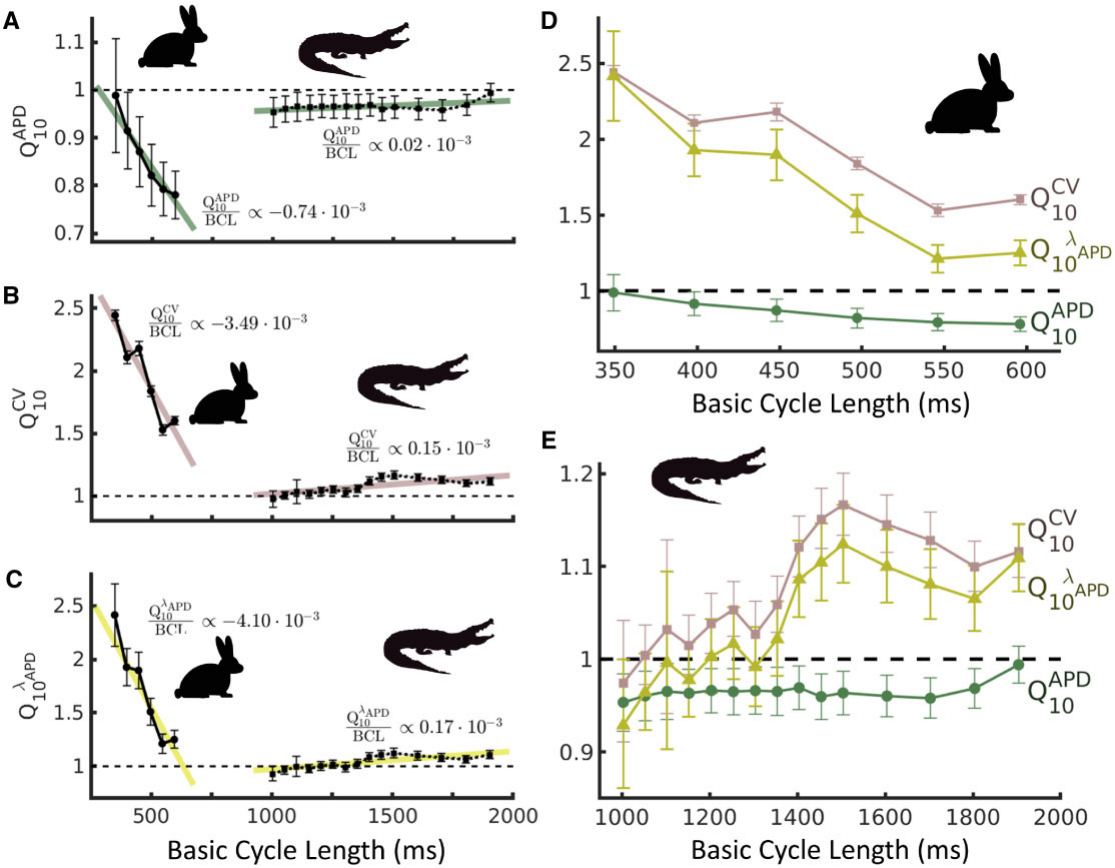
Temperature coefficients *Q*_10_ for cardiac dynamic properties in rabbit and alligator across BCLs for which hearts could be stimulated at both 23°C and 38°C. (**A**) The *Q*_10_ of APD in alligator approaches unity across all BCLs. In rabbit, APD is more greatly affected by the temperature at larger BCLs. (**B**) CV in rabbit is increasingly sensitive to temperature as BCL is reduced, and CV in alligator shows small temperature sensitivity across all BCLs. (**C**) Action potential wavelength $\lambda_{\text{APD}}$ in alligator is insensitive to temperature across all BCLs. This wavelength in rabbit exhibits strong temperature sensitivity, exacerbated further as BCL is reduced. (**D** and **E**) *Q*_10_ for all coefficients overlaid for each species. In rabbit (**D**), *Q*${}_{10}^{\text{CV}}$ and *Q*${}_{10}^{\text{APD}}$ become increasingly unbalanced at shorter BCLs, whereas in alligator (**E**), temperature sensitivities of APD and CV coordinate and preserve a temperature-insensitive $\lambda_{\text{APD}}$ across BCLs. Error bars denote 1 SD. Colored traces in **A**–**C** denote linear regressions with slopes listed in plots.

The *Q*_10_ values for cardiac wave dynamics were calculated for each animal (shown in Fig. 4 for a single rabbit and alligator; others are provided in Supplementary Material) as a function of BCL across the range for which hearts at both 23°C and 38°C could be stimulated, with SD *δ*Q_10_ calculated in terms of the SDs *δ*K${}_{T_{1}}$ and *δ*K${}_{T_{2}}$ as

$$\delta Q_{10}=Q_{10}\left(\frac{10^{{}^{\circ}}C}{T_{2}-T_{1}}\right)\sqrt{{\left(\frac{\delta K_{T_{1}}}{K_{T_{1}}}\right)}^{2}+{\left(\frac{\delta K_{T_{2}}}{K_{T_{2}}}\right)}^{2}}.$$

## Results

### Susceptibility to VF

All rabbit hearts exhibited VF at 38°C when the BCL of stimulation was shorter than ∼150 ms. When the temperature was reduced in rabbit hearts to 23°C, VF occurred for BCLs as large as 340 ms. No alligator hearts were observed to fibrillate under any circumstances. BCLs in alligator hearts were reduced until reaching the VFRP, at which point stimulations could not elicit excitations. The VFRPs in alligators were ∼860 and 1000 ms at 38°C and 23°C, respectively. Attempts to initiate VF in alligator hearts using the standard 9 V battery protocol (Bishop et al. 2014) consistently failed, including during *in situ* manipulations (data not shown).

### Action potential morphology and propagation

Temperature reduction in the rabbit heart substantially increased APD and decreased CV, in agreement with prior studies (Fedorov et al. 2008). When action potentials from all measured BCLs and both animals are compared (Fig. 1A and B), the two species exhibit clear differences in action potential morphology beyond APD. At both temperatures depolarization in the alligator action potential was observed to last ∼100 ms, ∼5 times the duration seen in the rabbit, for which depolarization elapsed within 20 ms. Beat-to-beat alternation in action potential morphology, or alternans, may be observed by overlaying successive action potentials as in Fig. 1C and D. While alternans occurred at both temperatures in the rabbit for short BCLs, alternans magnitude increased prominently at 23°C and over a larger range of BCLs consistent with other mammalian studies (Laurita and Rosenbaum 2008; Filippi et al. 2014). Alligators did not exhibit alternans regardless of temperature or BCL.

Tissue-scale dynamics in the alligator were mostly unaffected by temperature but were even more sensitive in the rabbit. Snapshots during the propagation of an action potential from apex to base, shown in Fig. 2, accentuate the visibly appreciable differences in the temperature sensitivity of CV. Furthermore, the increased wavefront curvature observed in the hypothermic rabbit heart implies an increased anisotropy ratio, which has been correlated with an increased risk of arrhythmia (Fedorov et al. 2008).

At all BCLs measured at both temperatures, the rabbit heart experienced an increase in APD ranging from 5% to 50% when the temperature was reduced from 38°C to 23°C (Fig. 3A, left), whereas the alligator only experienced between 5% and 10% increase in APD when the temperature was reduced (Fig. 3A, right). Temperature had some effect on reducing CV in the alligator (Fig. 3B); however, while apex-to-base CV decreased by 30–67% in rabbits, alligators experienced a 20% reduction at most.

### Action potential wavelength

Although temperature reduction from 38°C to 23°C likewise reduced action potential wavelengths $\lambda_{\text{APD}}$ at corresponding BCLs in each species (Fig. 3C), the minimum achieved action potential wavelength was unchanged (3 ± 1 cm) in the rabbit but increased (6 ± 1 cm to 10 ± 1 cm) in the alligator. At both temperatures, the onset of VF in rabbits occurred at BCLs for which $\lambda_{\text{APD}}$ became comparable to heart size (3 cm, dotted horizontal line). At both temperatures, the large VFRP in the alligator maintained a $\lambda_{\text{APD}}$ far greater than the length of the heart.

### Temperature coefficients

Calculations of *Q*_10_ (see Fig. 4) show that the temperature sensitivities of cardiac wave parameters in the alligator are low, with averages of *Q*${}_{10}^{\text{APD}}=0.97\pm 0.10$ and *Q*${}_{10}^{\text{CV}}=1.08\pm 0.23$. Interestingly, these temperature sensitivities were largely independent of BCL as well. Linear regressions across BCLs (Fig. 4A– C, colored lines) show that a 1000 ms change in BCL translates to changes of 0.02 (*Q*${}_{10}^{\text{APD}}$, R${}^{2}=31\%$) and 0.15 (*Q*${}_{10}^{\text{CV}}$, R${}^{2}=53\%$). Furthermore, these changes were complementary, such that the temperature sensitivity of action potential wavelength averaged over all BCLs was low (*Q*${}_{10}^{\;\lambda_{\text{APD}}}=1.04\pm 0.26$), and its linear regression shows a change in 0.17 for every 1000 ms changed in BCL (*R*${}^{2}=65\%$). In contrast, rabbits showed much more sensitive CV, with an average *Q*${}_{10}^{\text{CV}}=1.95\pm 0.16$, while APD appeared less sensitive (*Q*${}_{10}^{\text{APD}}=0.86\pm 0.19$). Both, however, were strongly affected by BCL, showing changes of 0.74 (*Q*${}_{10}^{\text{APD}}$, R${}^{2}=93\%$) and 3.49 (*Q*${}_{10}^{\text{CV}}$, *R*${}^{2}=91\%$) over a 1000 ms change in BCL according to best fit regression. As a result, the temperature sensitivity of action potential wavelength was large (*Q*${}_{10}^{\;\lambda_{\text{APD}}}=1.70\pm 0.59$) and strongly dependent on BCL, changing in value by 4.10 across a 1000 ms change in BCL (*R*${}^{2}=86\%$).

## Discussion

The crocodilian heart serves as an interesting example of ectothermic anatomy and physiology. Apart from a fully divided ventricle (Beddard and Mitchell 1895), which may be a vestige of the endothermic past in crocodilian ancestors (Hillenius and Ruben 2004; Seymour et al. 2004), they retain the reptilian style outflow tract which allows for a pulmonary bypass shunt (Jones and Shelton 1993; Eme et al. 2009). Compared to other reptiles of similar size, they also appear to be more restricted in their maximal heart rates (Joyce et al. 2018; Boukens et al. 2019). Recent investigations into the alligator’s cardiac electrophysiology have likewise revealed the existence of a conduction pathway for electrical excitations, reminiscent of the atrioventricular conduction system found in birds and mammals (Jensen et al. 2018) which depends on the formation of a complete ventricular septum during development (Kvasilova et al. 2020). And although electrocardiographic deflections in alligators vary across temperatures in a manner similar to heterothermic endotherms (Wilber 1960; Huggins et al. 1969; Fedorov et al. 2008; Egorov et al. 2012; Jensen et al. 2018), there are distinct differences which are not attributable to temperature or conduction system alone (Boukens et al. 2019). How these differences in cardiac electrical properties vary simultaneously across both temperature and cardiac cycle has before now been unknown.

APD and CV in the rabbit at 38°C are well matched to maintain a safe value for $\lambda_{\text{APD}}$ for all but the shortest of BCLs. And although the rabbit heart permits stimulation at these shorter BCLs (or equivalently, higher heart rates), there is a trade-off with an increased risk of cardiac arrhythmia as $\lambda_{\text{APD}}$ quickly reduces to a critical length. Alligator hearts resisted stimulation at these short BCLs, and values of $\lambda_{\text{APD}}$ were maintained well above dangerous levels.

Reducing temperature from 38°C to 23°C generally decreases CV and increases APD in the hearts of both alligators and rabbits, but the magnitude of these effects in the alligator are considerably lower and more similar to each other, which further helps safeguard the alligator from cardiac electrical arrhythmias. The high temperature sensitivity of CV in rabbit hearts greatly impacts susceptibility to arrhythmia, as the action potential wavelength $\lambda_{\text{APD}}$ becomes dangerously short at a much larger BCL in 23°C than in 38°C, providing a deadly substrate for functional reentry and fibrillation (Allessie et al. 1976, 1977). In contrast, functional and electrical dynamics in alligator hearts remain coordinated due to similar *Q*_10_ values of relevant variables across the entire range of measured BCLs. This coordination in the alligator is visible in Fig. 4 (right), where the temperature sensitivities of APD (A) and CV (B) maintain a balanced insensitivity of $\lambda_{\text{APD}}$ (C) across BCLs. Alternatively, the rabbit (Fig. 4, left) demonstrates the consequences of a mismatched balance in temperature sensitivities. Although the opposing effects of temperature on APD and CV reasonably preserve $\lambda_{\text{APD}}$ at the largest of BCLs, there is no coordination as BCL is reduced. The trade-off in the rabbit to permit stimulation at shorter BCLs becomes increasingly dangerous as the reduction of temperature further exacerbates risk of $\lambda_{\text{APD}}$ approaching a critical length.

The alligators’ large VFRP prevented stimulation at the short BCLs achievable in the rabbit, and the increase in VFRP at 23°C more than compensates for any increased risk of arrhythmia introduced by changes in APD or CV. Consequently, the alligator’s maximum heart rate is nearly an order of magnitude lower than that of the rabbit, severely limiting peak cardiac output and the ability of the circulatory system to support elevated aerobic metabolism. This relationship underlies the fundamental trade-off between the species: alligators can function at a wide range of heart temperatures without risk of cardiac arrhythmia but are restricted to lower heart rates, while rabbits can achieve high heart rates necessary to sustain an active and endothermic metabolism but are critically vulnerable to temperature changes.

This thermal insensitivity and large VFRP in the alligator heart are not traits shared by all ectotherms. For example, cardiac wave properties in zebrafish (Rayani et al. 2018), frogs (Mashima and Matsumura 1964; Goto et al. 1976, 1978), and turtles (Stecyk et al. 2007) show significant thermal sensitivity. In the case of zebrafish, maintaining wave parameters across temperatures and cycle lengths is unnecessary since their small hearts preclude any risk of reentry-based arrhythmia. And although larger species with low heart rates (large BCLs) do not risk reentry during healthy function, the absence of a sufficiently large VFRP can permit fibrillation in the event of an unfortunately timed ectopic beat. The susceptibility to fibrillation observed in frogs (Savino and Valentinuzzi 1988) and turtles (Hoffman et al. 1951) serves to illustrate the threatening alternative in this trade-off.

The sensitivity of the domestic rabbit heart to low temperature may be a consequence of its stable core body temperature in all conditions. Although rabbits do not hibernate, a variety of other mammals do enter periods of decreased metabolic rate and body temperature, and consequently these species exhibit an assortment of antiarrhythmic measures in hypothermic conditions, including a consistent $\lambda_{\text{APD}}$ and increased VFRP (Duker et al. 1983; Johansson 1996; Fedorov et al. 2008; Egorov et al. 2012). Similarly, non-hibernating but poikilothermic mammals, such as sloths, are better at resisting induced VF (Oliveira et al. 1980; Valentinuzzi et al. 1984). The similar properties we have observed in the alligator heart likewise enable survival over a wide range of temperatures, suggesting that the fundamental mechanism either has been retained from archosaur ancestors or has been lost in modern birds. Detailed characterization of heart electrophysiology in other reptile species is needed to answer the evolutionary history of arrhythmic properties. The fundamental trade-off with heart rate is irrelevant to ectothermic species, due to their reliance on anaerobic metabolism for “bursts” of high performance, making sustained high heart rates superfluous even when active (Bennett 1982; Seymour 2013; Joyce et al. 2018).

More broadly, these results highlight the perils of temperature-induced mismatch between interacting, dynamic processes, and the need for robust solutions. With minimal cardiac innervation, the propagation of electrical waves across heart muscle is driven entirely by intrinsic biochemical processes which, combined with the ability of these waves to interact, leaves cardiac dynamics uniquely vulnerable to disruption by temperature-induced mismatch of reaction rates (Fenton et al. 2013; Filippi et al. 2014). It remains uncertain to what extent the nervous system may be able to compensate for these mismatches in other muscular systems which show strong temperature effects, such as locomotion and feeding, particularly since the nervous system itself may be vulnerable to temperature-induced changes (Montgomery and Macdonald 1990), causing performance loss in tasks requiring coordination (Greenwald 1974).

## Supplementary Material

obaa047_Supplementary_Data

## Supplementary data

Supplementary data available at *IOB* online.
