# Supplementary material for: Defibrillate You Later, Alligator: Q10 Scaling and Refractoriness Keeps Alligators from Fibrillation
Source: Integr Org Biol. 2021 Jan 27;3(1):obaa047. doi: 10.1093/iob/obaa047 (PMC8101277; doi:10.1093/iob/obaa047)
Supplement: obaa047_Supplementary_Data [file obaa047_supplementary_data.zip › obaa047_Supplementary_Data/arabic_abstract.docx]

أزل الرجفان لاحقًا أيها التمساح: تحجيم ومقاومة معامل درجة الحرارة Q10 يمنعان حدوث الرجفان في التماسيح

يعتمد الانقباض القلبي الفعال خلال كل نبضة قلب على تنسيق موجة كهربية من الإثارة تنتشر عبر القلب، وقد يؤدي انتشار الموجات غير المتجانسة المستحث ديناميكيًا إلى الانكسار وبدء عدم انتظام ضربات القلب الناتجة عن ظاهرة إعادة الدخول، حيث تؤدي الموجات الكهربائية سريعة الدوران إلى إثارة ذاتية متكررة تقوض وظائف القلب وقد تؤدي إلى الموت القلبي المفاجئ. إن الأنواع الحيوانية التي تعمل بفعالية على مدى واسع من درجات حرارة القلب يجب أن توازن بين العديد من العمليات الكيميائية الحيوية المتفاعلة والحساسة لدرجة الحرارة من أجل الحفاظ على انتشار الموجة الطبيعي في جميع درجات الحرارة. لاستكشاف كيفية تجنب هذه الأنواع للحالات الخطرة عبر درجات الحرارة، قمنا برسم خريطة ضوئية للنشاط الكهربائي عبر أسطح قلوب التماسيح (*Alligator mississippiensis*) عند 23 درجة مئوية و 38 درجة مئوية على نطاق من معدلات ضربات القلب الفسيولوجية ومقارنتها مع تلك الخاصة بالأرانب (*Oryctolagus cuniculus*) . لقد وجدنا أنه على عكس الأرانب، تظهر التماسيح تغييرات طفيفة في معاملات الموجة (مدة السيالة العصبية وسرعة التوصيل) التي تكمل بعضها البعض للاحتفاظ بأطوال موجية فيزيولوجية كهربية مماثلة عبر درجات الحرارة وترددات السرعة. تستوعب الفيزيولوجيا الكهربية للقلب في الأرانب معدلات ضربات القلب المرتفعة اللازمة للحفاظ على التمثيل الغذائي النشط والممتص للحرارة على حساب زيادة خطر عدم انتظام ضربات القلب والضعف الشديد للتغيرات في درجات الحرارة، في حين أن ضربات القلب في التمساح تسمح بوظيفة فعالة على مدى درجات حرارة القلب دون وجود خطورة حدوث حالة من عدم انتظام ضربات القلب الكهربائية مثل الرجفان، ولكن تبين أن الأمر يقتصر على معدلات ضربات القلب المنخفضة.
