# Supplementary material for: Defibrillate You Later, Alligator: Q10 Scaling and Refractoriness Keeps Alligators from Fibrillation
Source: Integr Org Biol. 2021 Jan 27;3(1):obaa047. doi: 10.1093/iob/obaa047 (PMC8101277; doi:10.1093/iob/obaa047)
Supplement: obaa047_Supplementary_Data [file obaa047_supplementary_data.zip › obaa047_Supplementary_Data/danish_abstract.docx]

Defibrillator dig senere, alligator: Q10 skalering og refraktorering holder alligatorer fra at fibrilatorerer

Effektiv hjertemuskel sammentrækning i løbet af hvert hjerteslag beror på koordination mellem en elektrisk bølge af spændning som strømmer ud over hjerted. Dynamisk induserede heterogene bølge strømme kan frakturere og påbegynde en gentagende arrytmisk hjerte funktion, under hvilke hurtigt roterende elektriste bølger fører til genfølgende selv-spændinger som kompromiterer hjerte funktionen og potentielt resulterer i pludselig død fra hjerte-stop. Arter som fungerer effektivt over en stor rækkevidde af temperaturer må balancere de mange interaktive, temperatur-sensitive biokemikalske processer for at at vedligeholde normal bølge formering ved alle temperaturer. For at undersøge hvordan disse arter undgår farlige stater over temperaturene, har vi optisk kortlagt de elektriske aktiviteter under huden på alligator (*Alligator mississippiensis*) hjerter ved 23°C og 38°C over en række af fysiologiske hjete rytmer og sammenligned dem med kaniners (*Oryctolagus cuniculus*). Vi ser at alligatorer adskiller sig fra kaniner da de udviser minimal variering i bølge parametrene (aktion potentiel varighed og konduktions hastighed) som komplimenterer hinanden for at bibeholde lignende elektrofysiologiske bølgelængder over temperaturer og pacing frekvenser. Hjerte elektrofysiologien hos kaniner rummer de høje hjerterytmer der er nødvendige for at bibeholde aktivitet og endotermisk metabolisme, men på bekostning af højned risiko for hjerte arytmi og kritisk sårbarhed for temperatur ændringer, hvorom alligatorer har mulighede for effektiv funktion over en række hjerte temperaturer uden risiko for elektrisk hjerte arytmier så som fibrilatorer, men begrænset til lave hjerte rytmer.
