# Supplementary material for: Defibrillate You Later, Alligator: Q10 Scaling and Refractoriness Keeps Alligators from Fibrillation
Source: Integr Org Biol. 2021 Jan 27;3(1):obaa047. doi: 10.1093/iob/obaa047 (PMC8101277; doi:10.1093/iob/obaa047)
Supplement: obaa047_Supplementary_Data [file obaa047_supplementary_data.zip › obaa047_Supplementary_Data/french_abstract.docx]

Pas besoin de défibriller votre alligator; Le factor Q10 et la période réfractaire du potentiel d’action empêchent les alligators de fibriller.

La contraction cardiaque efficace repose sur la coordination d'une onde électrique d'excitation qui se propage à travers le cœur (le potentiel d’action). La propagation de ces ondes peut se déstabiliser dynamiquement et déclencher des arythmies cardiaques. Ces dernières se caractérisent par des circuits de réentrée, au cours desquelles des ondes électriques à rotation rapide conduisent à une auto-excitation répétée. Cela compromet la fonction cardiaque et peut entraîner une mort cardiaque subite.

Les espèces animales qui fonctionnent le mieux sur une large plage de températures doivent équilibrer les nombreux processus biochimiques qui interagissent et qui sont sensibles à la température. De cette manière, ils maintiennent une propagation normale des ondes cardiaques à toutes les températures. Pour étudier comment différentes espèces évitent les problèmes liés aux variations de températures, nous avons cartographié l'activité électrique présente à la surface des cœurs d'alligator « *Alligator mississippiensis* » à 23°C et 38°C sur une plage de fréquences cardiaques physiologiques. Nous avons comparé les résultats avec la même expérience réalisée sur des lapins « *Oryctolagus cuniculus* ».

Nous constatons que contrairement aux lapins, les ondes de potentiel d’action chez les alligators présentent seulement des changements minimes (durée du potentiel d'action et vitesse de conduction). Ces modifications se compensent pour conserver des longueurs d'onde électrophysiologiques similaires à travers les différentes températures et fréquences de stimulation. Au contraire, l'électrophysiologie cardiaque des lapins s'accommode des fréquences cardiaques élevées nécessaires pour maintenir un métabolisme actif et endothermique au prix d'un risque accru d'arythmie cardiaque. Qui plus est, les lapins possèdent une vulnérabilité accrue aux changements de température, alors que celle des alligators permet un fonctionnement efficace sur une gamme de températures cardiaques sans risque de déclencher des arythmies électriques cardiaques telles que la fibrillation.
