# Supplementary material for: Defibrillate You Later, Alligator: Q10 Scaling and Refractoriness Keeps Alligators from Fibrillation
Source: Integr Org Biol. 2021 Jan 27;3(1):obaa047. doi: 10.1093/iob/obaa047 (PMC8101277; doi:10.1093/iob/obaa047)
Supplement: obaa047_Supplementary_Data [file obaa047_supplementary_data.zip › obaa047_Supplementary_Data/german_abstract.docx]

Der Alligator ohne Defibrillator — Q10-Skalierung und der Refraktärzustand schützen Alligatoren vor Herzflimmern

Eine effektive Herzkontraktion während jedes Herzschlags beruht auf der Koordination einer elektrischen Erregungswelle, die sich über das Herz ausbreitet. Die dynamisch induzierte heterogene Wellenausbreitung kann jedoch aufbrechen und Herzrhythmusstörungen auslösen. Hierbei kommt es zu einem Wiedereintritt (reentry) bei dem schnell rotierende elektrische Wellen zu wiederholter Selbstanregung führen, die die Herzfunktion beeinträchtigt und unter Umständen zum plötzlichen Herztod führt. Tierarten, die für einen großen Bereich von Herztemperaturen auf eine stabile und effektive Herzfunktion angewiesen sind, müssen die vielen wechselwirkenden, temperaturempfindlichen biochemischen Prozesse steuern, um eine normale Wellenausbreitung bei allen Temperaturen aufrechtzuerhalten. Um zu untersuchen, wie diese Arten gefährliche Herzzustände über einen breiten Temperaturbereich hinweg vermeiden, haben wir die elektrische Aktivität an der Oberfläche von Alligatorherzen (*Alligator mississippiensis*) bei 23°C und 38°C in einem Bereich physiologischer Herzfrequenzen optisch sichtbar gemacht und die Resultate mit entsprechenden Messungen an Kaninchenherzen (*Oryctolagus cuniculus*) verglichen. Wir stellten fest, dass Alligatoren im Gegensatz zu Kaninchen minimale Änderungen der Wellenparameter (Aktionspotentialdauer und Ausbreitungsgeschwindigkeit) aufweisen, die sich so ergänzen, dass über Temperaturen und Stimulationsfrequenzen hinweg ähnliche elektrophysiologische Wellenlängen auftreten. Die Herzelektrophysiologie von Kaninchen trägt den hohen Herzfrequenzen Rechnung, die zur Aufrechterhaltung eines aktiven und endothermen Stoffwechsels erforderlich sind, auf Kosten eines erhöhten Risikos für Herzrhythmusstörungen und einer kritischen Anfälligkeit für Temperaturänderungen. Die Elektrophysiologie der Alligatoren hingegen ermöglicht eine effektive Herzfunktion über einen weiten Bereich von Herztemperaturen ohne das Risiko elektrischer Herzrhythmusstörungen wie Flimmern, ist dabei jedoch auf niedrige Herzfrequenzen beschränkt.
