# Supplementary material for: Defibrillate You Later, Alligator: Q10 Scaling and Refractoriness Keeps Alligators from Fibrillation
Source: Integr Org Biol. 2021 Jan 27;3(1):obaa047. doi: 10.1093/iob/obaa047 (PMC8101277; doi:10.1093/iob/obaa047)
Supplement: obaa047_Supplementary_Data [file obaa047_supplementary_data.zip › obaa047_Supplementary_Data/greek_abstract.docx]

«Απινίδωσέ μετά, αλιγάτορα: η κλιμάκωση Q10 και η ανυποταξία κρατούν τους αλιγάτορες από τη μαρμαρυγή.»

Η αποτελεσματική καρδιακή συστολή σε κάθε χτύπο της καρδιάς βασίζεται στον συντονισμό ενός ηλεκτρικού κύματος διέγερσης που διανέμεται στην καρδιά. Η δυναμικά επαγόμενη διάδοση ετερογενών κυμάτων μπορεί να σπάσει και να ξεκινήσει με επανεισαγωγή καρδιακών αρρυθμιών, κατά τη διάρκεια των οποίων γρήγορα περιστρεφόμενα ηλεκτρικά κύματα οδηγούν σε επαναλαμβανόμενη αυτοδιέγερση που θέτει σε κίνδυνο την καρδιακή λειτουργία και μπορεί να οδηγήσει σε ξαφνικό καρδιακό θάνατο. Γένη που λειτουργούν αποτελεσματικά σε μεγάλο εύρος καρδιακής θερμοκρασίας πρέπει να εξισορροπούν τις πολλές αλληλεπιδρούσες, ευαίσθητες στη θερμοκρασία βιοχημικές διεργασίες για να διατηρήσουν τη φυσιολογική διάδοση των κυμάτων σε όλες τις θερμοκρασίες. Για να διερευνήσουμε πώς αυτά τα γένη αποφεύγουν επικίνδυνα επίπεδα θερμοκρασιών, χαρτογραφήσαμε οπτικά την ηλεκτρική δραστηριότητα στις επιφάνειες των καρδιών αλιγάτορα (*Alligator mississippiensis*) στους 23°C και 38°C σε μια σειρά φυσιολογικών καρδιακών παλμών και τα συγκρίναμε με αυτά των κουνελιών (*Oryctolagus cuniculus*). Βρήκαμε ότι σε αντίθεση με τα κουνέλια, οι αλιγάτορες εμφανίζουν ελάχιστες αλλαγές στις παραμέτρους κυμάτων (διάρκεια δυναμικής δράσης και ταχύτητα αγωγιμότητας) που αλληλοσυμπληρώνονται για να διατηρήσουν παρόμοια ηλεκτροφυσιολογικά μήκη κύματος σε θερμοκρασίες και συχνότητες βηματοδότησης. Η καρδιακή ηλεκτροφυσιολογία των κουνελιών προσαρμόζεται στους υψηλούς καρδιακούς παλμούς που απαιτούνται για τη διατήρηση ενός ενεργού και ενδοθερμικού μεταβολισμού με το κόστος αυξημένου κινδύνου καρδιακής αρρυθμίας και κρίσιμης ευαισθησίας σε αλλαγές της θερμοκρασίας, ενώ αυτή των αλιγατόρων επιτρέπει αποτελεσματική λειτουργία σε ένα εύρος καρδιακών θερμοκρασιών χωρίς κίνδυνο καρδιακών ηλεκτρικών αρρυθμιών όπως η μαρμαρυγή, αλλά περιορίζεται σε χαμηλούς καρδιακούς ρυθμούς.
