# Supplementary material for: Defibrillate You Later, Alligator: Q10 Scaling and Refractoriness Keeps Alligators from Fibrillation
Source: Integr Org Biol. 2021 Jan 27;3(1):obaa047. doi: 10.1093/iob/obaa047 (PMC8101277; doi:10.1093/iob/obaa047)
Supplement: obaa047_Supplementary_Data [file obaa047_supplementary_data.zip › obaa047_Supplementary_Data/hungarian_abstract.docx]

 A Q10 méretarány és refrakteresség megakadályozza az aligátorok esetében a fibrilláció fellépését. A minden szívveréshez szükséges megfelelő szív összehúzódás feltétele, az elektromos hullám átvezetése a szíven. A dinamukusan gerjesztett heterogén hullám propagáció megtörhet, amely hozzájárulhat a reentry alapú aritmiához. Ilyen esetben a gyorsan forgó elektromos hullámok ismételt öngerjesztéshez vezetnek, amely veszélyezteti a szívműködést és potenciálisan hirtelen szívhalált eredményezhet. Azon fajok esetében, amelyek a szív hőmérsékletének nagy tartományában hatékonyan működnek, egyensúlyban kell tartaniuk a sok egymással kölcsönhatásban lévő, hőmérsékletre érzékeny biokémiai folyamatot a normál állapot fenntartása érdekében a lehetséges hőmérsékleti intervallum egészében. Annak megvizsgálására, hogy ezek a fajok hogyan kerülik el a veszélyes állapotokat különböző hőmérsékletek esetén, optikailag feltérképeztük az elektromos aktivitást aligátorok (*Alligator mississippiensis*) szívének felszínén 23°C-on és 38°C-on, a fiziológiás pulzusszám tartományában, és összehasonlítottuk a nyulak (*Oryctolagus cuniculus*) esetében tapasztalt értékekkel. Megállapítottuk, hogy a nyulaktól eltérően az aligátorok minimális változásokat mutatnak a hullámparaméterekben (akció potenciál időtartama és vezetési sebessége), amelyek úgy állnak összhangban, hogy hasonló elektrofiziológiai hullámhosszakat tartsanak fenn a hőmérsékletek és az ingerlési frekvenciák között. A nyulak szívelektrofiziológiája alkalmazkodik az aktív és endoterm anyagcsere fenntartásához szükséges magas pulzusszámhoz a szívritmuszavarok fokozott kockázatának és a hőmérsékleti változásokkal szembeni kritikus sérülékenysége árán, míg az aligátorok hatékony működést tesznek lehetővé a szív hőmérsékletének számos tartományában a szívritmuszavarok által okozott kockázatok – így például  fibrilláció nélkül, de csak alacsony pulzusszám esetén.
