# Supplementary material for: Defibrillate You Later, Alligator: Q10 Scaling and Refractoriness Keeps Alligators from Fibrillation
Source: Integr Org Biol. 2021 Jan 27;3(1):obaa047. doi: 10.1093/iob/obaa047 (PMC8101277; doi:10.1093/iob/obaa047)
Supplement: obaa047_Supplementary_Data [file obaa047_supplementary_data.zip › obaa047_Supplementary_Data/italian_abstract.docx]

E poi defibrilla un alligatore; refrattorietà e scala Q10 precludono gli alligatori dalla fibrillazione

Una contrazione efficace durante ogni battito cardiaco si basa sulla coordinazione di un’onda elettrica di eccitazione che propaga attraverso il cuore. Una propagazione ondosa irregolare indotta dinamicamente può fratturarsi ed indurre aritmie cardiache tramite rientri, durante i quali si riscontrano rapide onde elettriche rotanti che inducono auto-eccitazioni ripetute che compromettono la funzione cardiaca e risultano, potenzialmente, nella morte cardiaca improvvisa. Le specie che vivono efficacemente su un ampio intervallo di temperature cardiache devono bilanciare i molteplici processi biochimici interagenti e sensibili alla temperatura per mantenere una propagazione ondosa normale a tutte le temperature. Per investigare come queste specie evitino stati rischiosi alle varie temperature, abbiamo mappato otticamente l’attività elettrica attraverso le superfici del cuore dell’alligatore (*Alligator mississippiensis*) a 23°C e 38°C coprendo un intervallo di ritmi cardiaci fisiologici e li abbiamo confrontati con quelli dei conigli (*Oryctolagus cuniculus*). Riscontriamo che diversamente dai conigli, gli alligatori mostrano cambiamenti minimali nei parametri ondosi (durata del potenziale d’azione e velocità di conduzione) che si complementano lun l’altro per mantenere lunghezze d’onda elettrofisiologiche similari tra le varie temperature e frequenze di stimolazione. L’elettrofisiologica cardiaca dei conigli accomoda le elevate frequenze necessarie per supportare un metabolismo sostenuto ed endotermico al costo di un alto rischio di aritmia cardiaca e vulnerabilità critica alle variazioni termiche, laddove quella degli alligatori permette un funzionamento efficace su un ampio intervallo di temperature del cuore senza rischio di aritmie elettriche come la fibrillazione, sebbene sia limitata a basse frequenze cardiache.
