# Supplementary material for: Defibrillate You Later, Alligator: Q10 Scaling and Refractoriness Keeps Alligators from Fibrillation
Source: Integr Org Biol. 2021 Jan 27;3(1):obaa047. doi: 10.1093/iob/obaa047 (PMC8101277; doi:10.1093/iob/obaa047)
Supplement: obaa047_Supplementary_Data [file obaa047_supplementary_data.zip › obaa047_Supplementary_Data/mandarin_abstract.docx]

心臟跳動的過程中，有效的心臟收縮有賴於協調性的電興奮波傳播。 當動態中產生的異源性電興奮波傳播時，有可能分裂而誘發迴返性心律不整，此時快速的迴旋性電興奮波導致重複的心肌自我刺激，有害於心臟功能甚至造成心肌突然死亡。有些動物的心臟必須在溫差很大的環境下運作，這類的動物必須在許多對溫度敏感的生化途徑之間做取捨，以維持在各種溫度下心臟正常電興奮波的傳播。為了研究動物如何避免溫差造成的危險狀態，我們使用光學方法測量了密河鱷 (*Alligator mississippiensis*) 在23 ^o^C 和 38 ^o^C 以及一系列生理性心率下的心臟電生理活性，並與穴兔 (*Oryctolagus cuniulus*) 的結果相對照。我們發現，不同於穴兔的是，密河鱷的電興奮波參數 （包括動作電位的持續時間和傳導速率）在溫度和心率改變時只有些微的差異並彼此互補，致使在不同溫度與刺激頻率下，電生理的波長仍能維持相似。穴兔的心臟電生理則容許快速的心率，如此才能維持牠高活性的內溫性代謝，但是如此的代價是增加心律不整的危險以及對溫度改變耐受力脆弱。而密河鱷的電生理特性使牠的心臟能夠在較大的溫度變化範圍內有效工作，而且不會有心律不整（例如顫動）的危險， 唯有心率較慢的限制。
