# Supplementary material for: Defibrillate You Later, Alligator: Q10 Scaling and Refractoriness Keeps Alligators from Fibrillation
Source: Integr Org Biol. 2021 Jan 27;3(1):obaa047. doi: 10.1093/iob/obaa047 (PMC8101277; doi:10.1093/iob/obaa047)
Supplement: obaa047_Supplementary_Data [file obaa047_supplementary_data.zip › obaa047_Supplementary_Data/polish_abstract.docx]

„Tu defibrylator nie pomoże, aligatorze! Skalowanie współczynnika Q10 i oporność zapobiegają migotaniu serca u aligatorów”

Skuteczne skurcze mięśnia sercowego przy każdym biciu serca polegają na koordynacji elektrycznej fali wzbudzenia przechodzącej przez serce. Dynamicznie wywołana heterogeniczna propagacja fali może się załamać i zainicjować sercowe arytmie oparte na ponownym wstąpieniu, podczas których szybko krążące fale elektryczne prowadzą do ponownego samowzbudzenia, co z kolei kompromituje funkcje serca i może potencjalnie spowodować nagła śmierć sercową. Gatunki, które skutecznie funkcjonują w szerokim zakresie temperatur serca, muszą równoważyć wiele wrażliwych na temperaturę procesów biochemicznych, aby zachować normalną propagacje fali we wszystkich temperaturach. Aby zbadać jak takie gatunki unikają niebezpiecznych stanów związanych ze zmianą temperatury, zmapowaliśmy optycznie elektryczną aktywność na powierzchni serca u aligatora (*Alligator mississippiensis*) w temperaturach 23°C i 30°C w całym zakresie fizjologicznego tętna oraz porównaliśmy ją do aktywności w sercu królika (*Oryctolagus cuniculus*). Nasze wyniki wykazały, że w przeciwieństwie do królika, u aligatora występują minimalne zmiany w parametrach falowych (czasie trwania potencjału czynnościowego i prędkości przewodzenia), co pozwala zachować podobną elektrofizjologiczną długość fali w całym zakresie temperatur i częstotliwości stymulacji. Elektrofizjologia serca królika pozwala na wysokie tętno potrzebne do podtrzymania aktywnej i endotermicznej przemiany materii kosztem zwiększonego ryzyka arytmii serca i istotnej podatności na zmiany temperatury. U aligatorów, elektrofizjologia serca zezwala na skuteczną działalność przez cały zakres temperatur serca bez ryzyka arytmii takich jak migotanie, ale jednocześnie jest ograniczona do niskiego tętna.
