# Supplementary material for: Defibrillate You Later, Alligator: Q10 Scaling and Refractoriness Keeps Alligators from Fibrillation
Source: Integr Org Biol. 2021 Jan 27;3(1):obaa047. doi: 10.1093/iob/obaa047 (PMC8101277; doi:10.1093/iob/obaa047)
Supplement: obaa047_Supplementary_Data [file obaa047_supplementary_data.zip › obaa047_Supplementary_Data/russian_abstract.docx]

Масштабирование Q10 и рефрактерности предохраняют аллигаторов от фибрилляции

Эффективное сокращение сердца зависит от координации электрической волны возбуждения, распространяющейся по сердцу. Динамически индуцированное гетерогенное распространение волн может привести к разрыву и возникновению сердечных аритмий, связанных с повторным входом. Быстро вращающиеся электрические волны приводят к многократному самовозбуждению, которое ставит под угрозу сердечную функцию и потенциально приводит к внезапной сердечной смерти. Виды, которые эффективно функционируют в широком диапазоне температур сердца, должны уравновешивать множество взаимодействующих, чувствительных к температуре биохимических процессов, чтобы поддерживать нормальное распространение волн при всех температурах. Чтобы исследовать, как эти виды избегают опасных состояний при различных температурах, мы оптически  картировали электрическую активность  на поверхности сердец аллигатора *Alligator mississippiensis* при 23ºC и 38ºC в диапазоне физиологической частоты сердечных сокращений и сравнили их с таковой у кроликов *Oryctolagus cuniculus*. Мы обнаружили, что, в отличие от кроликов, аллигаторы демонстрируют минимальные изменения в параметрах волны (длительность потенциала действия и скорость проводимости), которые дополняют друг друга, сохраняя одинаковые электрофизиологические длины волн в зависимости от температуры и частоты стимуляции. Электрофизиология сердца кроликов учитывает высокую частоту сердечных сокращений, необходимую для поддержания активного и эндотермического метаболизма за счет повышенного риска сердечной аритмии и критической уязвимости к изменениям температуры. Сердце аллигаторов эффективно функционирует в диапазоне температур сердца без риска сердечных электрических аритмий, таких как фибрилляция, но ограничивается низкой частотой сердечных сокращений.
